# Supplementary material for: Seminoma and Embryonal Carcinoma Footprints Identified by Analysis of Integrated Genome-Wide Epigenetic and Expression Profiles of Germ Cell Cancer Cell Lines
Source: PLoS One. 2014 Jun 2;9(6):e98330. doi: 10.1371/journal.pone.0098330 (PMC4041891; doi:10.1371/journal.pone.0098330)
Supplement: File S1 — ZIP file containing DMRforPairs output for significant regions. Please start from the html files. (ZIP) [file pone.0098330.s008.zip › figures/15380.pdf]

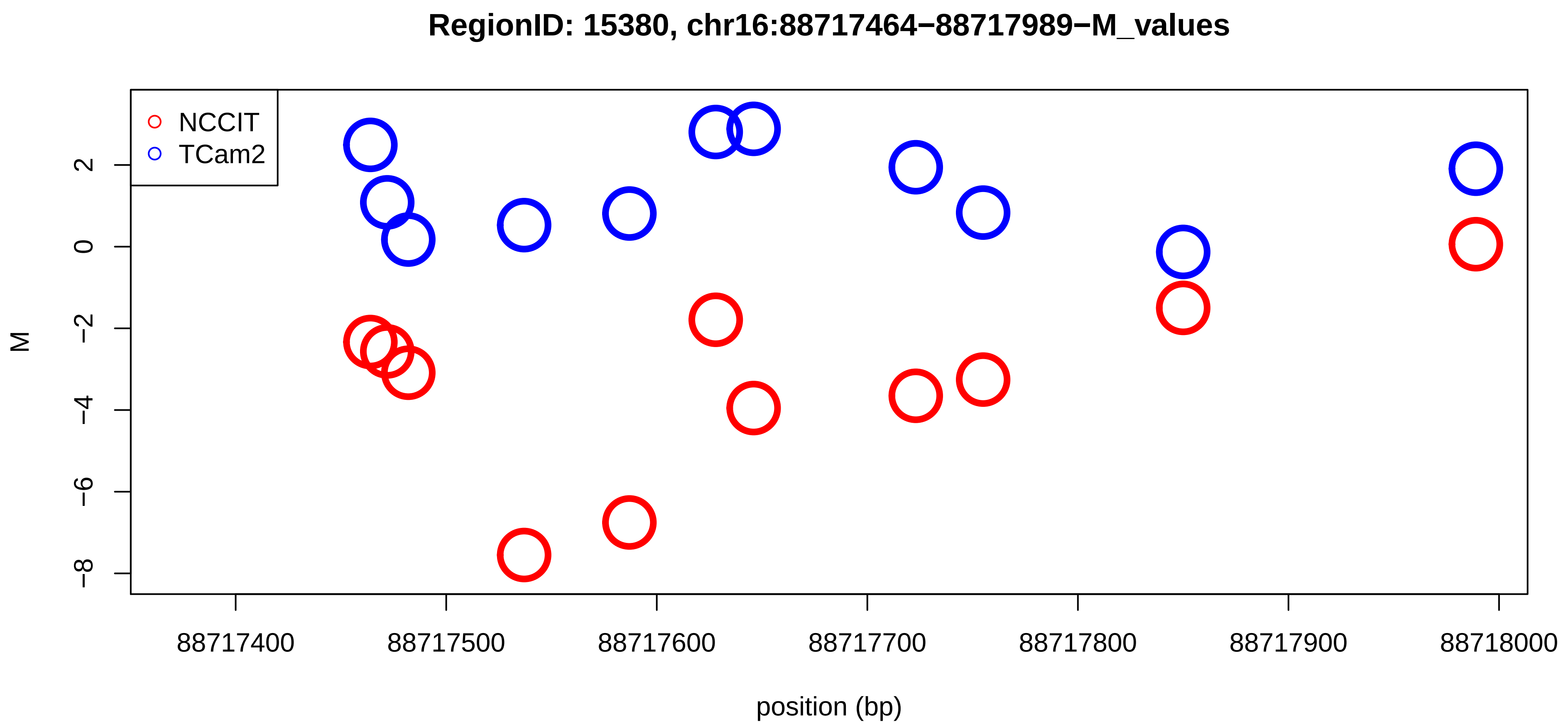
RegionID: 15380, chr16:88717464−88717989−M_values
NCCIT
TCam2
M
2
0
−2
−4
−6
−8
88717400
88717500
88717600
88717700
88717800
88717900
88718000
position (bp)

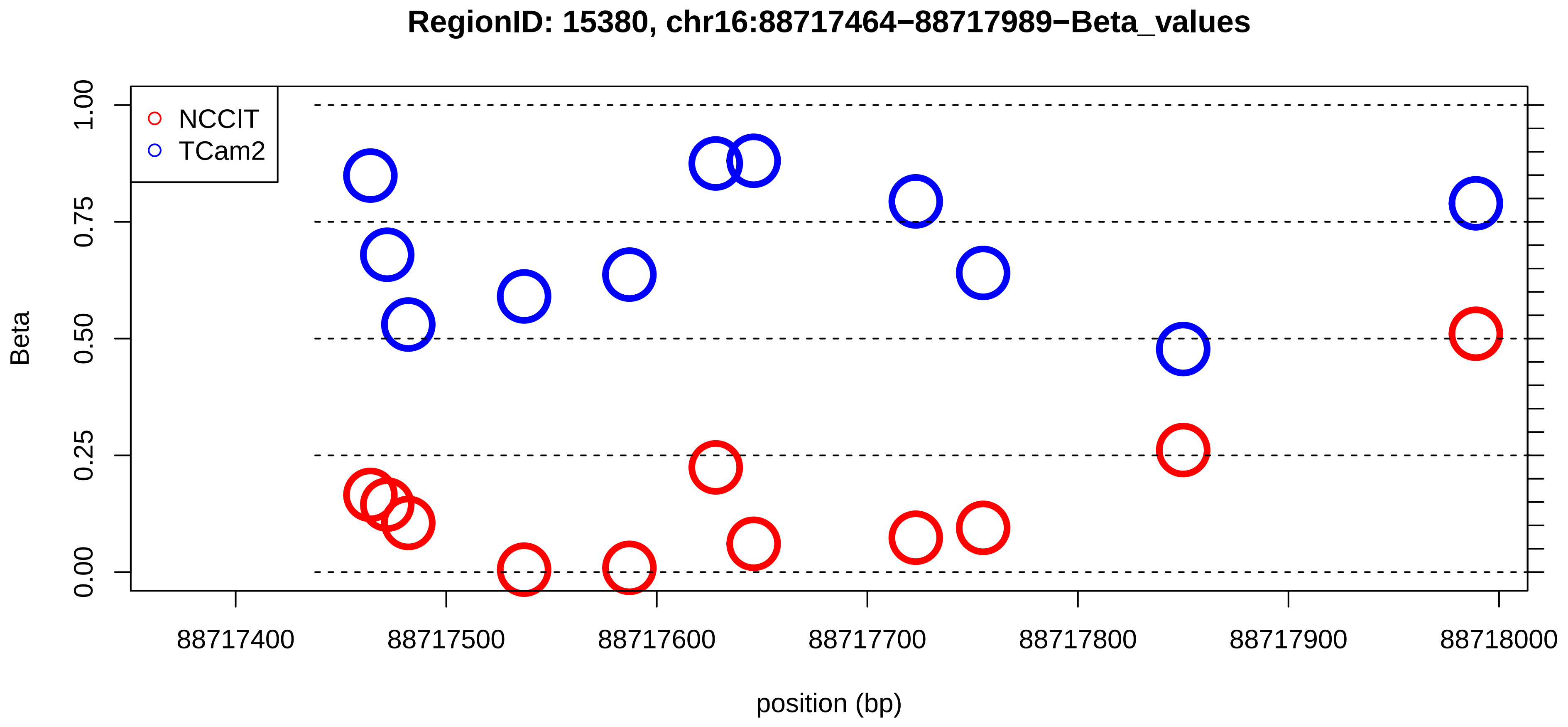
RegionID: 15380, chr16:88717464−88717989−Beta_values
NCCIT
TCam2
Beta
1.00
0.75
0.50
0.25
0.00
88717400
88717500
88717600
88717700
88717800
88717900
88718000
position (bp)

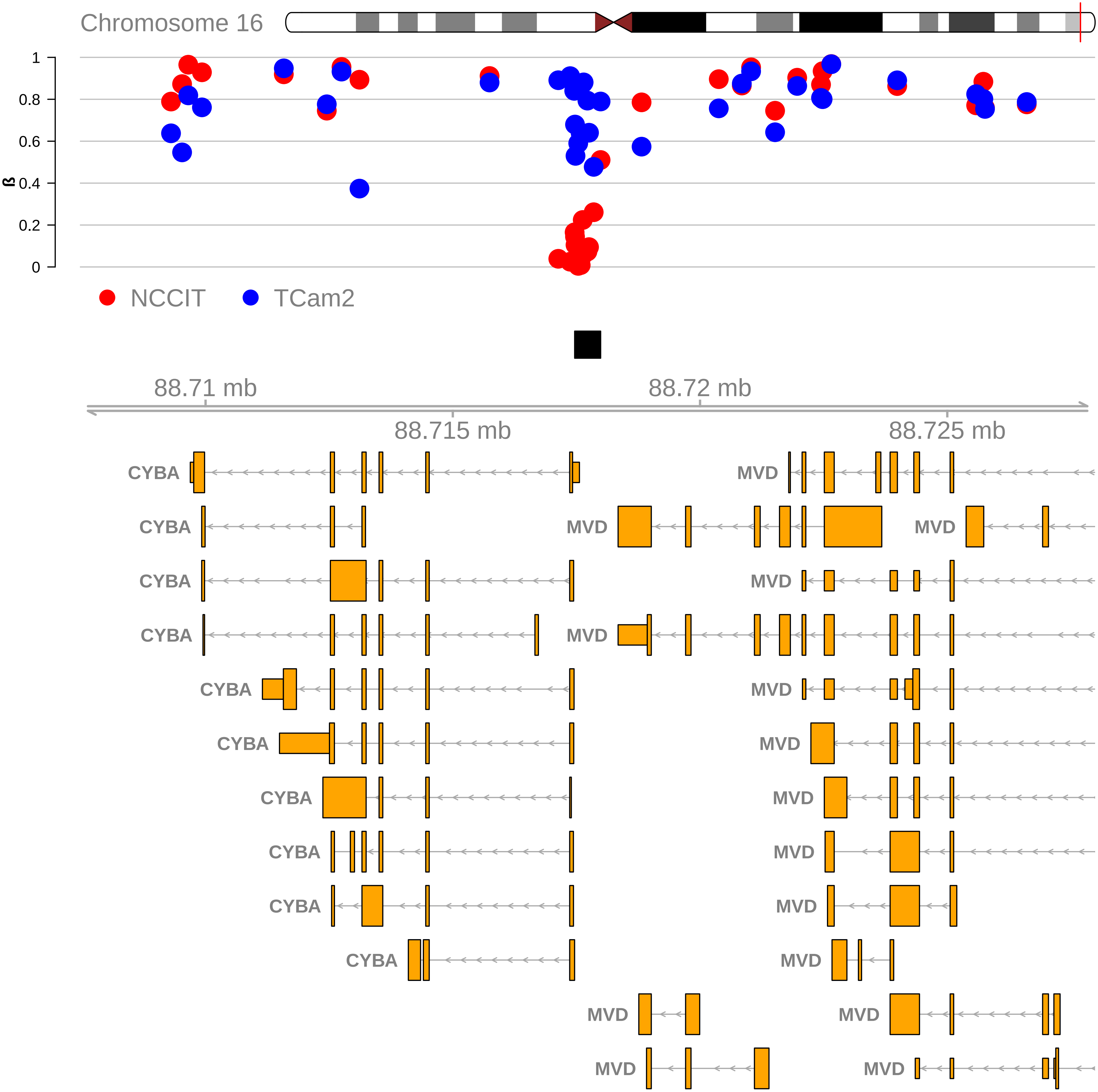

Chromosome 16
1
0.8
0.6
β 0.4
0.2
0
NCCIT
TCam2
88.71 mb
88.72 mb
88.715 mb
88.725 mb
CYBA
MVD
